# P100 Lymphoma—not the only malignancy in Sjögren’s syndrome

**DOI:** 10.1093/rap/rkag113.130

**Published:** 2026-11-03

**Authors:** Sabeen Malik, Spencer Ellis

**Affiliations:** Lister Hospital, Stevenage, United Kingdom; Lister Hospital, Stevenage, United Kingdom

## Abstract

**Introduction:**

We present a case which depicts broad spectrum of haematological malignancies which can occur in Sjögren’s syndrome. It shows rare association of T-cell large granular leukaemia with Sjögren’s, extending beyond more common association of B-cell lymphoma.

**Case description:**

A 79/F with a background of bronchiectasis, hypertension and aortic stenosis was first referred in 2016 with dry mouth, MCP joint swelling and triphasic Raynaud’s phenomenon.

On assessment, she had significant sicca symptoms with dry mouth, dry eyes and mild right parotid gland enlargement. Serology showed positive ANA and ENA (Ro positive, La negative and anti centromere antibodies positive >240), raised ESR and normal C3/4. Ultrasound showed synovitis in the right index MCP. A diagnosis of Sjogren’s syndrome with overlap connective tissue disease (CTD) features was made, and hydroxychloroquine was started.

Her disease course demonstrated persistent issues with sicca symptoms, Raynaud’s phenomenon and inflammatory arthritis with later diagnosis of seropositive rheumatoid arthritis (RF 70, CCP >340).

From 2019 onwards, she developed progressive neutropenia. Serial monitoring showed elevated dsDNA titres and complement levels remained normal.

In May 2024, the patient deteriorated with severe neutropenia, anaemia and elevated inflammatory markers. Bone marrow biopsy confirmed a diagnosis of T-cell large granular lymphocytic leukaemia. She was started on methotrexate and GCSF therapy with improvement in neutrophils. In 2025 CT CAP had shown splenomegaly.

Her planned TAVI for severe aortic stenosis was initially postponed but was successfully performed in December 2024.

The subsequent disease course was complicated by recurrent infections and methotrexate was discontinued following sepsis. This was associated with a flare of inflammatory arthritis with partial response to intramuscular steroids.

Sicca symptoms remained a significant issue but improved with pilocarpine.

Recent Status (2026)

By early 2026, her IA remained active following cessation of methotrexate, with documented synovitis. Blood tests showed fluctuating counts, including leucocytosis in the context of GCSF therapy and recent infection.

Hydroxychloroquine has been restarted and further DMARD being considered although this requires close liaison with haematology due to the risk of cytopenias in the setting of T LGLL.

**Discussion:**

This case shows several important teaching points:

1. Autoimmune disease can evolve over time, with initial features of Sjögren’s syndrome progressing to overlap with seropositive rheumatoid arthritis.

Interpretation of WCC monitoring is a challenge as autoimmune conditions can be linked to neutropenia. But in our case persistent neutropenia in absence of active disease features, led to further investigations, revealing underlying leukaemia. This highlights importance of keeping wide differential diagnosis and considering further investigation.

2. Sjögren’s syndrome has a well-documented association with B cell lymphoproliferative disorders such as MALT lymphoma or diffuse Large B cell lymphoma, whereas our patient developed T- cell Large Granular Lymphocytic Leukaemia.

Only a small number of cases describing an association between Sjögren’s syndrome and T-cell Large Granular Lymphocytic (T-LGL) leukaemia have been reported in the literature.

3. Finally, our case emphasises the importance of multidisciplinary care, including close communication between rheumatology, haematology and cardiology, particularly in the context of coexisting malignancy and major cardiac intervention.

**Key learning points:**

Haematological malignancy is strongly associated with Sjögren’s and rheumatoid arthritis.

1. While Sjögren’s is classically linked to B cell non-Hodgkin lymphoma, but it can also significantly elevate risk of T cell malignancies, as in this case.

Although the underlying mechanism between these two conditions remains unclear, STAT3 gene mutations have been suggested as a potential contributing pathway. Further research is required to better understand this association.

2. Persistent neutropenia can have multiple causes in patients with CTD who are treated with immunosuppressive agents. Where this is not well explained, alternative causes should be considered, and haematological investigation may be required.

3. Treatment decisions require balancing immunosuppression with infection risk, particularly in elderly patients with comorbidities.

4. Multidisciplinary management is essential in complex cases involving autoimmune disease and haematological malignancy.

